# Primary fallopian tube adenocarcinoma: A case report and review of the literature

**DOI:** 10.1016/j.ijscr.2022.107555

**Published:** 2022-08-31

**Authors:** Harrad Mouna, Watik Fedoua, Boufettal Houssine, Sakher Mahdaoui, Samouh Naïma

**Affiliations:** Department of Gynecology-Obstetrics, Ibn Rochd University Hospital, Casablanca, Morocco; Faculty of Medecine and Pharmacy, Hassan II University, Casablanca, Morocco

**Keywords:** Adenocarcinoma, Primary fallopian tube, Surgery, Chemotherapy

## Abstract

**Introduction:**

Primary cancer of the fallopian tube is very rare. The diagnosis is rarely made before surgery or histological study.

**Case report:**

We report the observation of a tubal adenocarcinoma in a 42-year-old female patient, discovered following an abdomino-pelvic mass. A total hysterectomy without adnexal preservation with omentectomy, appendectomy and partial bladder resection were performed, followed by platinum-based chemotherapy. Despite this observation, the authors report a review of the literature concerning the epidemiology, diagnosis, treatment and prognosis of this cancer.

**Discussion:**

Primary fallopian tube cancers are rare, representing 0.3 to 1.1 % of gynecological cancers. They are frequently confused with ovarian cancers in case of locally advanced disease and are clearly underestimated.

**Conclusion:**

The positive diagnosis is difficult because the clinical picture is polymorphic, MRI is of great diagnostic interest, the prognosis depends on the FIGO stage.

## Highlights

• Primary tubal cancer is very rare, frequently confused with ovarian cancers
• Abdomino-pelvic ultrasound is the key examination when a suspicious adnexal mass is found, which leads to pelvic MRI and, if necessary, surgical exploration
• The histological types are similar to those of the ovary, represented almost exclusively by epithelial tumors, 75% of which are high-grade serous carcinomas
• The therapeutic management of cancers of the tube is similar to that of the ovary and is based on the combination of maximum cytoreduction surgery and chemotherapy

## 1 Introduction

Primary fallopian tube cancer is very rare. Averages of 20 to 30 new cases are reported each year. The incidence of this cancer varies between 0.3 and 1.1 % among all gynecological cancers [1], [2]. We report a case of primary tubal adenocarcinoma in a 42-year-old woman, discovered following an abdomino-pelvic mass and treated by surgery and chemotherapy. In the light of this observation, the authors report a review of the literature concerning the epidemiology, diagnosis, treatment and prognosis of this cancer. All our work was reported in accordance with the SCARE criteria and guidelines [12].

## 2 Case report

**Fig. 1:**
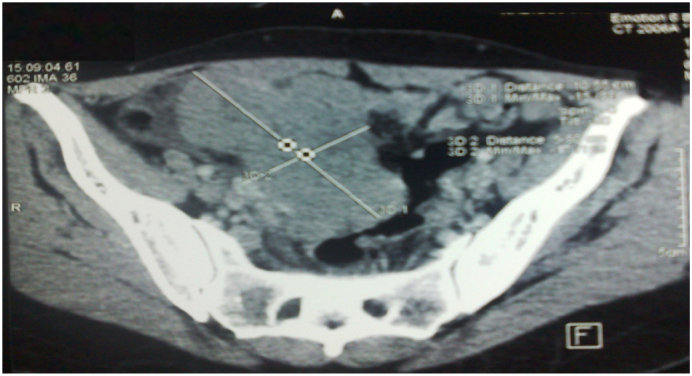
Abdominopelvic scan showing a pelvic-abdominal injury, tissue necrosis in places, slightly hand colored by the injection of contrast.

**Fig. 2:**
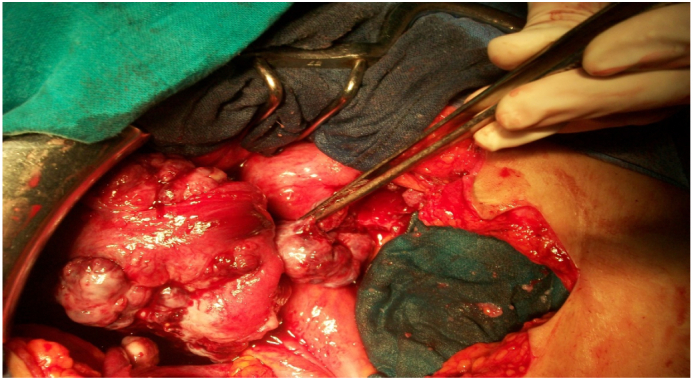
Preoperative view showing a solid tumor vegetating, measuring 100/80 mm of the right tube. Both ovaries and the contralateral horn were free of any macroscopic lesion.

**Fig. 3:**
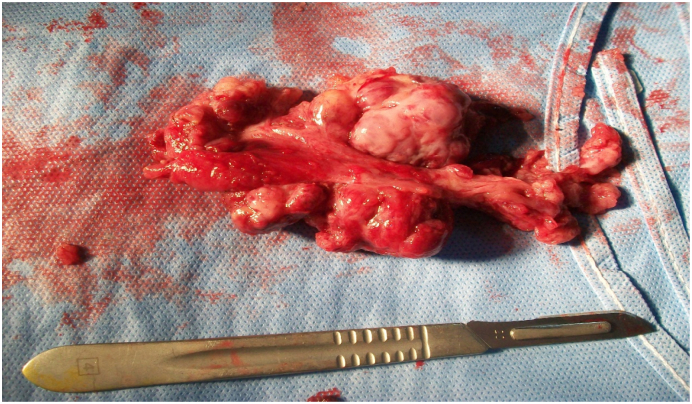
A tumor biopsy with the right tube tumor, sent in frozen section.

**Fig. 4:**
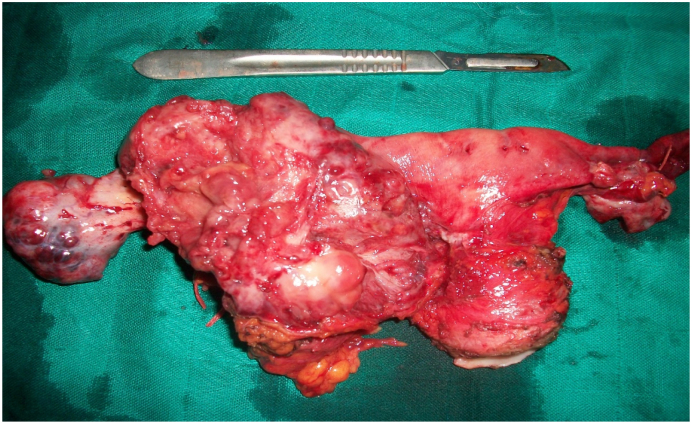
Surgical specimen of hysterectomy without conservation adnexal tumor showing tubal right. The two ovaries are macroscopically intact.

**Fig. 5:**
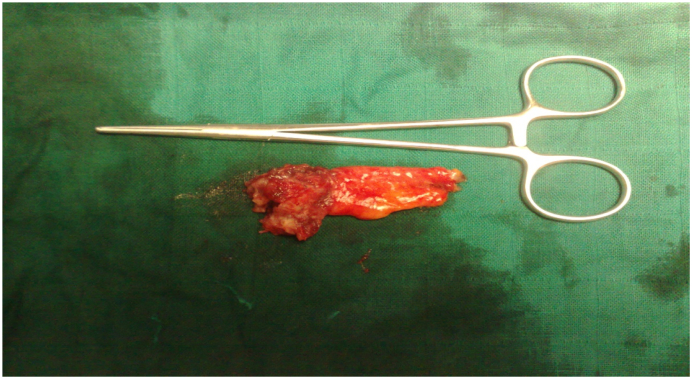
Room appendectomy showing the invasion of it by the tubal tumor.

Patient aged 42 years, single, with no personal or family history. She consulted for pelvic pain with a one-month-old pelvic mass. Examination revealed a hard abdominal-pelvic mass, measuring eight centimeters, which could not be displaced from the uterus. Ultrasound showed a solid, Doppler-vascularized mass measuring 112 × 85 mm. Abdominal-pelvic CT scan showed a patchy necrotic pelvic-abdominal tissue lesion, poorly enhanced by contrast injection, measuring 100 × 70 × 60 mm (Fig. 1). There were multiple peritoneal nodules with mesenteric fat over density as well as pelvic, lumbo-aortic, and inter-aortic-caval adenopathies. The liver and spleen were homogeneous and without focal lesions. Classification according to TNM 4C [3], CA 125 was increased to 708 IU/mL. An exploratory laparotomy that lasted 90 min performed by our team showed a solid vegetative tumor, measuring 100 × 80 mm, of the right tube. Both ovaries and the contralateral tube were free of lesions (Fig. 2). The tumor invaded the bladder and vermicular appendage. An ascites of 150 ml was aspirated. A biopsy of the tumor measuring 80 × 70 × 20 mm was sent for extemporaneous examination (Fig. 3). The result of this biopsy showed a poorly differentiated and invasive malignant tumor proliferation whose origin could only be defined after fixation and kerosene inclusion. A total hysterectomy with ovarectomy, appendectomy, omentectomy, peritoneal biopsies, and partial bladder resection were performed (Fig. 4, Fig. 5). The postoperative course was straightforward. The patient was discharged on the 15th day. The pathological diagnosis showed a solid, whitish tumor proliferation infiltrating the right tube, extending to the mesotubal area, adhering to the right lateral aspect of the uterine body and measuring 12 cm in diameter. It was consistent with a serous adenocarcinoma. The left adnexa, uterine body, cervix and omentum were free of tumor. A chest X-ray was normal. After a six-month follow-up, no recurrence was found.

## 3 Discussion

Primary fallopian tube cancers are rare, representing 0.3 to 1.1 % of gynecological cancers [1]. They are frequently confused with ovarian cancers in case of locally advanced disease and are clearly underestimated. They are unilateral in 85 % of cases [2], with an average of 20 to 30 new cases reported each year [3].

The fallopian tube is an organ with low oncogenic potential, which contrasts with its vulnerability to infection. Its prognosis is generally poor [4].

This disease affects middle-aged patients with a peak incidence at 50–60 years of age and rarely affects young or very old women. Some risk factors have been suggested such as infertility, chronic salpingitis or nulliparity, although no clear etiology has been demonstrated [5]. Mutations in the BRCA1 and BRCA2 genes are associated with a relative risk of 120 of developing tubal cancer. Thus, an oncogenetic opinion is most often sought. Tubal cancer is also associated with overexpression of p53, HER-2/neu and c-myc [2].

The clinical picture is constituted by the pathognomonic triad of Latzko or “Hydropstubaeprofluens” associating abdomino-pelvic pain, vaginal discharge (metrorrhagia, leucorrhoea or haematorrhoea) and a pelvic mass [6].

Primary cancers of the tube are distinguished from ovarian cancers by their more noisy symptomatology, with pain appearing rapidly due to early tubal distension. Metrorrhagia with negative endometrial curettage is also a warning sign [2].

In the end, these early and noisy clinical signs mean that tubal localization has a better prognosis than ovarian localization because it is discovered more quickly.

The first paraclinical examination to be requested is ultrasound, which often shows the same appearance as epithelial carcinomas of the ovary in the form of a mixed adnexal mass, solid and cystic, hypervascularized on Doppler [5], [6]. Pelvic MRI is used for staging; the typical appearance of a tubal carcinoma is manifested by a hyperintense T2-weighted signal and a hypointense T1-weighted signal with a solid appearance. Tumor markers, mainly Ca125, are sensitive but nonspecific and are often elevated. [7], [8]

Staging is based on ovarian cancer [4].

Treatment is also identical to the management of ovarian cancer, but their prognosis is better because they are most often diagnosed at an earlier stage[9]. The gold standard surgery is total hysterectomy, bilateral adnexectomy, omentectomy, pelvic and lomboaortic curage. Chemotherapy combining cisplatin and paclitaxel has the same indications as for ovarian cancer. Radiotherapy is abandoned because of its low efficacy. [10], [11]

The prognosis depends on the size of the tumor, the stage, the invasion, and the existence of a macroscopic postoperative residue [4]. The prognostic role of genetic alteration of p53, Kras, c-erb-2 and CA125 immunostaining of the tumor is being evaluated [4].

The overall 5-year survival of tubal malignancies is estimated at 44 % according to large series [9], [10], [11].

## 4 Conclusion

Tubal adenocarcinoma is a rare entity, of unknown etiology, underestimated or often confused with ovarian pathology with which it shares the same treatment and staging, the positive diagnosis is difficult because the clinical picture is polymorphic, MRI brings a great diagnostic interest, the prognosis depends on the FIGO stage.

## Provenance and peer review

Not commissioned, externally peer-reviewed.

## Consent

Written informed consent was obtained from the patient for publication of this case report and accompanying images. A copy of the written consent is available for review by the Editor-in-Chief of this journal on request.

## Ethical approval

I declare on my honor that the ethical approval has been exempted by my establishment.

## Sources of funding

None.

## Guarantor

Dr. watik fedoua

## Registration of research studies

None.

## Annals of medicine and surgery

The following information is required for submission. Please note that failure to respond to these questions/statements will mean your submission will be returned. If you have nothing to declare in any of these categories then this should be stated.

## CRediT authorship contribution statement

WATIK Fedoua:Corresponding author writing the paper

HARRAD Mouna: writing the paper

SAID HASNAA :writing the paper

BOUFETTAL Houssein: correction of the paper

MAHDAOUI Sakher: correction of the paper

BOUHYA Said : correction of the paper

## Declaration of competing interest

The authors declare having no conflicts of interest for this article.
